# Therapeutic peptides in gerontology: mechanisms and applications for healthy aging

**DOI:** 10.3389/fragi.2026.1790247

**Published:** 2026-04-07

**Authors:** Volodymyr Mavrych, Inna Shypilova, Olena Bolgova

**Affiliations:** 1 College of Medicine, Alfaisal University, Riyadh, Saudi Arabia; 2 School of Medicine, St. Matthew’s University, George Town, Cayman Islands

**Keywords:** aging, anti-aging interventions, epitalon, gerontology, growth hormone, healthspan, metabolic aging, neuroprotection

## Abstract

**Background:**

Peptide therapeutics represent an emerging frontier in gerontological medicine, targeting fundamental hallmarks of aging including metabolic dysfunction, telomere attrition, tissue repair impairment, and hormonal decline.

**Objective:**

To comprehensively review the mechanisms, clinical applications, evidence base, and safety profiles of therapeutic peptides with demonstrated or potential applications in healthy aging and age-related conditions.

**Methods:**

A comprehensive narrative review was conducted through systematic searches of PubMed, Scopus, and regulatory databases (FDA, WADA) from inception through January 2026. Search terms included “peptide therapeutics,” “aging,” “gerontology,” “healthspan,” combined with specific peptide names (tirzepatide, epitalon, GHK-Cu, BPC-157, TB-500, Semax, CJC-1295, ipamorelin, bremelanotide). Peer-reviewed articles, clinical trials, regulatory documents, and preclinical studies were evaluated. A total of 20 primary sources were selected based on relevance, methodological quality, and contribution to understanding peptide mechanisms and clinical outcomes in aging populations.

**Results:**

Nine peptides were identified spanning diverse aging interventions: metabolic restoration (tirzepatide), telomere biology (epitalon), dermal regeneration (GHK-Cu), tissue repair (BPC-157, TB-500), neuroprotection (Semax), growth hormone modulation (CJC-1295, ipamorelin), and sexual function (bremelanotide). FDA-approved agents demonstrated robust safety profiles from large-scale trials. Non-approved peptides showed promising preclinical and limited clinical evidence but lack long-term safety data and systematic validation. Significant knowledge gaps include optimal dosing regimens, combination therapy effects, and biomarkers for monitoring efficacy.

**Conclusion:**

Therapeutic peptides offer mechanistically diverse approaches to multiple aging hallmarks. While FDA-approved agents demonstrate clinical potential, investigational peptides require rigorous validation through well-designed clinical trials to establish safety and efficacy for healthspan extension.

## 1 Introduction

The global demographic shift toward an aging population has intensified the search for interventions that can extend healthspan (the period of life free from significant age-related disease and disability) rather than merely increasing lifespan. Peptide therapeutics have emerged as promising agents in gerontological medicine, targeting multiple hallmarks of aging, including genomic instability, telomere attrition, epigenetic alterations, loss of proteostasis (protein homeostasis), mitochondrial dysfunction, and cellular senescence (López-Otín et al., 2023). Unlike traditional small-molecule drugs, peptides offer high specificity for their molecular targets, potentially reducing off-target effects while addressing fundamental aging mechanisms (Fosgerau and Hoffmann, 2015).

This review examines nine peptides with demonstrated or potential applications in age-related conditions: tirzepatide, epitalon, GHK-Cu, BPC-157, TB-500, Semax, CJC-1295, ipamorelin, and bremelanotide (PT-141). These agents represent diverse approaches to aging intervention, from FDA-approved therapeutics to investigational compounds, each targeting distinct but interconnected aging pathways (Table 1). We organize these peptides into functional categories based on their primary mechanisms and clinical applications, then critically evaluate current evidence, safety considerations, and future directions for this emerging field.

**TABLE 1:** Therapeutic peptides - mechanisms, applications, and clinical evidence.

Peptide/References	Classification	Mechanism of action	Clinical applications	Key evidence	Regulatory status/Evidence level	Key safety concerns and evidence gaps
Tirzepatide (Mounjaro/Zepbound) (Thomas et al., 2021; Rosenstock et al., 2021; Frías et al., 2021; Jastre et al., 2022)	Dual GIP/GLP-1 receptor agonist	Activates glucose-dependent insulinotropic polypeptide and glucagon-like peptide-1 receptors; stimulates insulin secretion, reduces glucagon, and slows gastric emptying	Type 2 diabetes mellitus, obesity/weight management, obstructive sleep apnea	Phase 2b: HbA1c reduction up to 2.4% vs. placebo at 26 weeks (Thomas et al., 2021); SURPASS-1: mean reduction 2.07 percentage points from baseline vs. placebo (Rosenstock et al., 2021); SURPASS-2: mean reduction 2.30 percentage points from baseline (Frías et al., 2021); SURMOUNT-1: weight loss 15.0%–20.9% at 72 weeks (Jastre et al., 2022)	FDA-approved; Phase 3 RCTs (n > 7,700)	GI side effects; long-term CV outcomes ongoing
Epitalon (Epithalon) (Khavinson et al., 2003; Araj et al., 2025; Khavinson et al., 2012a; Khavinson et al., 2012b)	Tetrapeptide (Ala-Glu-Asp-Gly)	Activates telomerase enzyme, lengthens telomeres, modulates pineal gland function, and increases melatonin synthesis	Anti-aging, longevity enhancement, and circadian rhythm regulation	Animal studies: enhances lifespan by 12%–24% in rodent models. Clinically: improved visual function in patients with retinitis pigmentosa and increased melatonin production	Not approved; small clinical studies	No long-term safety data; no Western RCTs
GHK-Cu (Copper Tripeptide) (Pickart and Margolina, 2018; Pickart et al., 2015)	Naturally occurring tripeptide-copper complex (Glycyl-L-histidyl-L-lysine-Cu^2+^)	Copper chelation enhances enzymatic activity; stimulates collagen/elastin synthesis, promotes angiogenesis, and modulates 31% of human genes	Skin regeneration, wound healing, anti-aging, hair growth, tissue repair	Gene modulation studies: upregulates antioxidant defense and DNA repair pathways; Plasma levels decline with age (200 ng/mL at age 20–80 ng/mL at age 60)	Not approved (cosmetic use); *in vitro*/animal	No clinical trials; systemic safety unknown
BPC-157 (Chang et al., 2011; Gwyer et al., 2019; Lee and Padgett, 2021; Lee et al., 2024; Lee and Burgess, 2025)	Synthetic pentadecapeptide (15 amino acids)	Activates VEGFR2 and NO pathways via Akt-eNOS axis; promotes angiogenesis, enhances ERK1/2 signaling, increases VEGF and EGR-1 expression	Tendon/ligament repair, muscle healing, gastrointestinal ulcer healing, wound repair	Animal studies: accelerated healing in tendons, ligaments, muscle, and GI tract; Human pilot studies: 58% sustained pain relief >6 months (knee pain pilot, n = 58); 83.3% complete symptom resolution (interstitial cystitis pilot, n = 12)	Not approved; pilot studies only (n = 12–58)	WADA-banned; theoretical tumor angiogenesis risk
TB-500 (Goldstein and Kleinman, 2015; Sosne and Kleinman, 2015; Wang et al., 2021)	Synthetic thymosin β4 fragment (Ac-LKKTETQ, 7 amino acids)	Actin-binding protein regulating cytoskeletal dynamics; enhances cell migration, promotes angiogenesis, releases anti-inflammatory acSDKP peptide	Muscle/tendon recovery, wound healing, tissue repair, cardiac protection, inflammation reduction	Animal studies: enhanced wound healing, muscle regeneration, cardiac function post-MI; Corneal studies: accelerated re-epithelialization	Not approved; Phase I safety only	WADA-banned; no efficacy RCTs in humans
Semax (Dolotov et al., 2006; Erickson et al., 2010; Mansour et al., 2025; Filippenkov et al., 2025; Gusev et al., 2018)	Synthetic heptapeptide, ACTH(4–10) analog (Met-Glu-His-Phe-Pro-Gly-Pro)	Increases BDNF (1.4-fold) and trkB phosphorylation (1.6-fold); modulates dopamine/serotonin systems; enhances neuroplasticity and antioxidant pathways	Cognitive enhancement, neuroprotection, stroke recovery, anxiety reduction	Animal studies: 3-fold increase in BDNF mRNA; Human stroke trials: improved neurological function and rehabilitation timing; Enhanced learning and memory performance	Not approved in West; Russian clinical trials	No independent Western validation
CJC-1295 (Teichman et al., 2006; Ionescu and Frohman, 2006; Alba et al., 2006; Malavige et al., 2021)	Synthetic GHRH analog with Drug Affinity Complex (DAC)	Binds GHRH receptors on pituitary somatotrophs; activates adenylyl cyclase/cAMP; stimulates pulsatile GH release; DAC provides albumin binding for extended half-life (6–8 days)	Growth hormone enhancement, anti-aging, lean muscle support, metabolic health	Single dose: 2-10 fold GH increase for 6+ days, 1.5-3 fold IGF-1 increase for 9–11 days; Preserved GH pulsatility; Animal studies: normalized growth with daily dosing; CJC-1295 combined with ipamorelin improved muscle strength	Not approved; Phase 2 discontinued	Trial death (unrelated); CV and cancer risk unknown
Ipamorelin (Raun et al., 1998; Malavige et al., 2021)	Selective GH secretagogue, pentapeptide (Aib-His-D-2-Nal-D-Phe-Lys-NH_2_)	Selective ghrelin receptor (GHS-R1a) agonist; stimulates GH release without affecting cortisol, prolactin, or ACTH (selective for GH at 200x ED_50_)	GH support, lean muscle development, fat loss, recovery enhancement	GH release potency similar to GHRP-6 but superior selectivity; No ACTH/cortisol elevation; Maintained selectivity at high doses	Not approved; preclinical + limited human	No long-term safety data
PT-141 (Bremelanotide) (Molinoff et al., 2003; Kingsberg et al., 2019; U.S. Food and Drug Administration, 2026d)	Cyclic heptapeptide, melanocortin receptor agonist (MC1R, MC3R, MC4R, MC5R)	Activates melanocortin receptors in CNS (MC3R/MC4R in hypothalamus/limbic system); enhances sexual desire through central dopamine modulation	Hypoactive sexual desire disorder (HSDD) in premenopausal women; erectile dysfunction (off-label)	Phase 3 women: +0.75 sexual encounters/month, reduced distress scores; Male studies: improvements in erectile function	FDA-approved June 2019; Phase 3 RCTs	Nausea, hypertension; approval limited to premenopausal women

This review is intended as a scientific overview for researchers and clinicians and does not constitute clinical recommendations.

## 2 Materials and methods

A comprehensive narrative review was conducted through systematic searches of PubMed, Scopus, and regulatory databases (FDA, WADA) from inception through January 2026. The full search string was: (“peptide therapeutics” OR “therapeutic peptides”) AND (“aging” OR “gerontology” OR “healthspan”) AND (“tirzepatide” OR “epitalon” OR “GHK-Cu” OR “BPC-157” OR “TB-500” OR “Semax” OR “CJC-1295” OR “ipamorelin” OR “bremelanotide” OR “PT-141”). Eligibility criteria included peer-reviewed primary studies and regulatory documents reporting mechanistic, preclinical, or clinical data in English. Peer-reviewed articles, clinical trials, regulatory documents, and preclinical studies were evaluated. The 20 primary sources were selected based on relevance, methodological rigor, and contribution to the evidence base for each peptide.

## 3 Metabolic and hormonal interventions

### 3.1 Tirzepatide: improving age-related metabolic parameters

Metabolic dysfunction represents a central feature of biological aging, characterized by progressive insulin resistance, dysregulated glucose homeostasis, accumulation of visceral adiposity, and increased risk of type 2 diabetes and cardiovascular disease (Palmer and Jensen, 2022). Tirzepatide, a dual glucose-dependent insulinotropic polypeptide (GIP) and glucagon-like peptide-1 (GLP-1) receptor agonist, improves metabolic parameters associated with age-related disease by restoring glycemic control and reducing adiposity in the context of type 2 diabetes and obesity. The peptide activates both GIP and GLP-1 receptors, stimulating insulin secretion, reducing glucagon release, and slowing gastric emptying (Thomas et al., 2021). Phase 2b studies demonstrated tirzepatide produces HbA1c reductions of up to 2.4% versus placebo at 26 weeks (Thomas et al., 2021), with Phase 3 SURPASS trials confirming mean HbA1c reductions from baseline of 2.07 percentage points with tirzepatide 15 mg (treatment difference vs. placebo: −2.11 percentage points; SURPASS-1) (Rosenstock et al., 2021) and 2.30 percentage points from baseline (treatment difference vs. semaglutide: −0.45 percentage points; SURPASS-2) (Frías et al., 2021) at the 15 mg weekly dose, producing clinically significant improvements in glycemic control in patients with type 2 diabetes, a condition prevalent in and associated with accelerated biological aging. Equally important from a gerontological perspective, the SURMOUNT trials demonstrated substantial weight loss of approximately 15%–20% at 72 weeks with tirzepatide, accompanied by visceral fat reduction and broad improvements in cardiovascular and metabolic risk factors, including blood pressure, fasting insulin, lipid profiles, and liver enzymes (Jastre et al., 2022).

Beyond glycemic and weight effects, tirzepatide may influence aging through multiple mechanisms. Visceral fat reduction decreases chronic low-grade inflammation (inflammaging), a key driver of age-related pathology (Franceschi and Campisi, 2014). Improved insulin sensitivity enhances cellular nutrient-sensing pathways, potentially mimicking some benefits of caloric restriction, the most robust intervention for lifespan extension in animal models (Madeo et al., 2019). FDA approval for type 2 diabetes (May 2022), obesity (November 2023), and obstructive sleep apnea (December 2024) established tirzepatide’s clinical utility, with typical dosing starting at 2.5 mg weekly and titrating to 5–15 mg based on response (U.S. Food and Drug Administration, 2026a; U.S. Food and Drug Administration, 2026b; U.S. Food and Drug Administration, 2026c).

### 3.2 Growth hormone axis restoration

Growth hormone (GH) secretion declines approximately 14% per decade after age 30, resulting in decreased lean body mass, increased adiposity (particularly visceral fat), reduced bone density, decreased skin thickness, and impaired recovery from illness or injury, a phenomenon termed “somatopause” (Toogood et al., 1996; Fernández-Garza et al., 2025). Two peptides offer distinct approaches to addressing this decline.

CJC-1295, a long-acting GHRH analog incorporating a Drug Affinity Complex (DAC) for albumin binding, addresses somatopause by restoring more youthful GH secretion patterns (Teichman et al., 2006). Unlike exogenous GH administration that suppresses endogenous production and eliminates pulsatility, CJC-1295 preserves physiological pulsatile GH release while increasing both pulse amplitude and trough levels (Ionescu and Frohman, 2006). Single CJC-1295 injections produced 2-10-fold increases in plasma GH sustained for 6+ days and 1.5-3-fold IGF-1 elevations lasting 9–11 days (Teichman et al., 2006). Animal studies suggest that daily CJC-1295 normalizes growth, body composition, and metabolic function in GH-deficient mice (Alba et al., 2006).

Ipamorelin complements CJC-1295 by stimulating GH release through ghrelin receptor activation rather than GHRH pathways (Raun et al., 1998). Its selectivity is remarkable, potent GH release without cortisol or prolactin elevation, even at doses 200-fold above the effective dose, avoiding the adverse endocrine effects seen with earlier GH secretagogues (Raun et al., 1998). The CJC-1295/ipamorelin combination is widely discussed in anti-aging medicine for its synergistic GH-stimulating effects through dual pathway activation. CJC-1295 combined with ipamorelin showed significantly improved maximum tetanic tension in murine models with glucocorticoid-induced muscle loss, but these findings are limited to animal studies (Malavige et al., 2021).

## 4 Cellular and molecular aging interventions

### 4.1 Epitalon: targeting telomere biology

Telomere attrition ranks among the most fundamental hallmarks of aging. These repetitive DNA sequences at chromosome ends shorten with each cell division, eventually triggering replicative senescence when critically short - a process underlying tissue dysfunction and organismal aging (Blackburn et al., 2015). Epitalon (Ala-Glu-Asp-Gly), a tetrapeptide derived from pineal gland extracts, represents the most extensively studied peptide targeting telomere biology (Khavinson et al., 2003).

Epitalon’s primary mechanism involves activating telomerase, the enzyme that adds telomeric repeats to chromosome ends. In human somatic cell cultures, epitalon treatment induces expression of the telomerase catalytic subunit (hTERT), increases enzymatic activity, and produces measurable telomere elongation sufficient to extend cellular lifespan beyond the Hayflick limit (Khavinson et al., 2003; Araj et al., 2025). Beyond telomerase activation, epitalon modulates multiple aging-related pathways. The peptide enhances pineal gland function and melatonin secretion, which decline markedly with age and contribute to circadian disruption, sleep disturbances, and increased oxidative stress (Araj et al., 2025; Khavinson et al., 2012a). Animal studies suggest that epitalon increases activity of antioxidant enzymes, including superoxide dismutase and glutathione peroxidase, bolstering cellular defenses against age-related oxidative damage, and extends median and maximum lifespan by 12%–24% in rodent longevity studies (Khavinson et al., 2012b).

Clinical trials have shown that Epitalon improves visual function in patients with Retinitis pigmentosa, with parabulbar injections of 5 µg per eye for 10 days, enhancing visual acuity, expanding peripheral fields, and reducing scotomas, without reported adverse effects (Araj et al., 2025). In a separate study, sublingual Epitalon (0.5 mg/day for 20 days) enhanced melatonin production and modulated circadian gene expression, including Clock, Cry2, and Csnk1e, suggesting that its geroprotective effects are mediated by restoring epiphyseal melatonin synthesis and circadian regulation (Araj et al., 2025).

### 4.2 GHK-Cu: epigenetic remodeling and dermal regeneration

Skin aging represents one of the most visible manifestations of biological aging, characterized by collagen loss, decreased elasticity, increased wrinkling, and impaired wound healing (Farage et al., 2013). Glycyl-L-histidyl-L-lysine complexed with copper (GHK-Cu) addresses multiple aspects of dermal aging through its multifaceted mechanism of action (Pickart and Margolina, 2018).

GHK-Cu stimulates collagen and elastin synthesis, the structural proteins that decline progressively with age. Gene profiling studies reveal GHK-Cu’s broad effects on cellular aging. The peptide modulates approximately 31% of human genes, upregulating pathways involved in antioxidant defense, DNA repair, and tissue remodeling while downregulating inflammatory and pro-fibrotic genes (Pickart and Margolina, 2018). This broad gene-expression modulation is associated with transcriptional profiles more characteristic of less-aged cells, as observed in vitro and dermal model systems; the functional significance of these changes *in vivo* remains to be established in controlled human studies (Pickart and Margolina, 2018; Pickart et al., 2015). Importantly, plasma GHK levels decline dramatically with age (from 200 ng/mL at age 20–80 ng/mL at age 60), directly correlating with decreased skin repair capacity and accelerated visible aging (Pickart and Margolina, 2018; Pickart et al., 2015).

## 5 Tissue repair and regeneration

Aging dramatically impairs tissue repair, contributing to increased recovery time from injuries, chronic non-healing wounds, and progressive musculoskeletal decline (Gould et al., 2015). Two peptides (BPC-157 and TB-500) show particular promise for enhancing tissue regeneration in aging contexts. The highest available human evidence for BPC-157 consists of small pilot studies (n = 12–58 patients).

### 5.1 BPC-157: enhanced healing in aged and damaged tissues

BPC-157, a 15-amino acid peptide derived from gastric protective proteins, promotes tissue repair through activation of angiogenic pathways (VEGFR2, Akt-eNOS, ERK1/2 signaling) essential for new blood vessel formation (Seiwerth et al., 2021). Aging tissues typically exhibit decreased vascularization and impaired angiogenic responses, contributing to poor healing (Ungvari et al., 2018). BPC-157s ability to enhance angiogenesis addresses this age-related deficit directly. Animal studies demonstrate accelerated healing of tendons, ligaments, muscles, and gastrointestinal tissues prone to age-related dysfunction (Chang et al., 2011; Gwyer et al., 2019). Gene expression studies show rapid upregulation (within 10 min) of multiple pro-regenerative genes, including VEGF, EGR-1, and various MAPK pathway components (Seiwerth et al., 2021).

Clinical evidence remains limited. Clinical pilot data derived from distinct populations indicated that 58% of elderly patients with chronic knee pain achieved sustained pain relief beyond 6 months following a single injection (Lee and Padgett, 2021), while 83.3% of middle-aged to elderly women with refractory interstitial cystitis experienced complete symptom resolution after a single intravesical dose (Lee et al., 2024). Although a small 2025 trial showed no adverse effects on cardiac or renal function following intravenous administration of 20 mg BPC-157, the peptide remains unapproved by the FDA for clinical use (Lee and Burgess, 2025).

### 5.2 TB-500: anti-inflammatory regulation in aged tissues

TB-500, derived from thymosin beta-4, functions primarily through actin regulation and anti-inflammatory mechanisms (Goldstein and Kleinman, 2015). Human evidence for TB-500 is limited to a single Phase I safety and pharmacokinetics trial. TB-500 is a synthetic fragment of thymosin β4 (residues 17–23, sequence Ac-LKKTETQ) and is not identical to full-length thymosin β4 or to recombinant human thymosin β4 used in clinical trials; findings from thymosin β4 studies should therefore not be uncritically extrapolated to TB-500. Peptide promotes cell migration to injury sites, enhances angiogenesis via VEGF upregulation, and releases anti-inflammatory mediators that modulate excessive inflammation, a particular problem in aged tissues where chronic low-grade inflammation impairs healing (Sosne and Kleinman, 2015). A Phase I clinical trial assessed the safety and pharmacokinetics of recombinant human thymosin beta-4 in healthy individuals. The study concluded that the peptide was well tolerated across single and multiple intravenous doses, with dose-proportional increases in plasma concentration and no significant accumulation after repeated administration (Wang et al., 2021).

## 6 Neuroprotection and quality of life

### 6.1 Semax: cognitive enhancement and neuroprotection

Cognitive decline represents one of the most feared aspects of aging, progressing from mild age-associated memory impairment to potentially devastating neurodegenerative diseases (Harada et al., 2013). Age-related structural and functional deterioration of the basolateral amygdala and medial prefrontal cortex (regions central to emotional memory, decision-making, and adaptive behavioral responses) contributes significantly to the cognitive and neuropsychiatric vulnerability observed in older adults (Mavrych et al., 2025). Semax, a synthetic heptapeptide derived from ACTH (4–10), addresses cognitive aging through multiple neuroprotective and cognitive-enhancing mechanisms (Dolotov et al., 2006).

Semax’s primary mechanism involves upregulation of brain-derived neurotrophic factor (BDNF), the master regulator of neuroplasticity that declines with age (Erickson et al., 2010). Administration of Semax produces 1.4-fold increases in hippocampal BDNF protein levels, 3-fold increases in BDNF mRNA expression, and enhanced activation of the BDNF receptor (trkB), collectively promoting neuronal survival, synaptic plasticity, and neurogenesis, processes that decline progressively with aging (Dolotov et al., 2006). Importantly, enhanced neuroplasticity through BDNF upregulation represents a critical defense against age-related neurovascular dysfunction and glymphatic impairment, which contribute significantly to cognitive decline and neurodegenerative processes (Mansour et al., 2025). Beyond BDNF modulation, Semax has been shown in animal ischemia models to modulate gene expression in neurotransmitter and inflammatory signaling pathways, and earlier pharmacological studies have described antioxidant and anti-neuroinflammatory properties (Dolotov et al., 2006; Filippenkov et al., 2025).

Clinical evidence from Russian studies demonstrates Semax’s utility in age-related cognitive conditions. Trials in elderly stroke patients showed improved neurological recovery, enhanced rehabilitation outcomes, and better cognitive function when Semax was added to standard care (Gusev et al., 2018). The standard regimen of Semax included 2 courses (6000 mcg/day) for 10 days, with a 20-day interval (Gusev et al., 2018).

### 6.2 Bremelanotide: addressing age-related sexual dysfunction

Sexual dysfunction increases dramatically with age in both sexes, driven by hormonal changes, vascular disease, medications, and psychosocial factors, significantly impacting quality of life and relationship satisfaction. Bremelanotide (PT-141) offers a mechanistically novel approach by targeting central melanocortin receptors (MC3R, MC4R) in hypothalamic and limbic regions rather than peripheral vascular mechanisms (Molinoff et al., 2003). This central action enhances sexual desire and arousal through modulation of dopaminergic pathways in brain regions controlling sexual motivation.

FDA approval (June 2019) for hypoactive sexual desire disorder (HSDD) in premenopausal women was supported by Phase 3 trials showing improved sexual desire, increased satisfying sexual encounters, and reduced distress (Kingsberg et al., 2019). While regulatory approval is limited to premenopausal women with HSDD, use in postmenopausal women and men has been described in the clinical literature, used off-label in some clinical settings; no population-level prevalence data are available. Studies in men demonstrated erectogenic effects, with improvements in erectile function, including in men with sildenafil-resistant erectile dysfunction (Molinoff et al., 2003). Standard dosing involves 1.75 mg subcutaneous injection 45 min before anticipated sexual activity, maximum once per 24 h and 8 times monthly (U.S. Food and Drug Administration, 2026d).

Complementing these pharmaceutical interventions, natural approaches to age-related hormonal decline, including dietary phytoestrogens, targeted micronutrients, gut microbiome modulation, strategic exercise programming, and bioactive compounds, offer evidence-based alternatives with favorable safety profiles for individuals with contraindications to or preferences against pharmacological hormone therapy (Bolgova et al., 2025; Shypilova et al., 2026).

## 7 Discussion

### 7.1 Different schools of thought

The application of peptides in gerontology reflects divergent philosophical approaches to aging intervention. The mainstream pharmaceutical approach, exemplified by tirzepatide and bremelanotide, emphasizes rigorous FDA approval pathways, large-scale randomized controlled trials, and treatment of specific age-related diseases (Lau and Dunn, 2018). This conservative approach prioritizes safety and regulatory compliance but may limit exploration of novel anti-aging interventions.

In contrast, the longevity medicine approach, prevalent in anti-aging clinics and compounding pharmacy networks, views aging itself as a modifiable process amenable to intervention (Kennedy et al., 2014). Practitioners in this paradigm use off-label peptides such as CJC-1295, ipamorelin, BPC-157, and TB-500 based on preclinical data and anecdotal clinical experience, accepting greater uncertainty in exchange for potential health-span benefits. This approach has enabled clinical experience with promising peptides but lacks systematic safety surveillance and efficacy validation.

A third perspective emerges from Russian gerontological research, which has developed and clinically used peptides such as epitalon and Semax for decades with apparent success (Khavinson et al., 2012b). These peptides have extensive clinical use in former Soviet states but lack independent Western validation, creating epistemological challenges for evidence evaluation.

### 7.2 Controversies and safety considerations

The peptide landscape in gerontology is fraught with controversy. FDA-approved agents (tirzepatide, bremelanotide) provide the highest quality safety and efficacy data from large-scale clinical trials involving thousands of participants (Jastre et al., 2022; Kingsberg et al., 2019). Tirzepatide safety was established in over 7,700 participants, with primarily gastrointestinal side effects (nausea, diarrhea, vomiting) being the most common adverse events, typically mild to moderate and diminishing with continued use (Rosenstock et al., 2021; Frías et al., 2021; Jastre et al., 2022).

Non-approved peptides present greater uncertainty. BPC-157 and TB-500, despite extensive preclinical data, lack adequate human clinical trials and long-term safety data (Gwyer et al., 2019). Theoretical concerns about angiogenic peptides potentially promoting tumor vascularization remain unaddressed, despite animal studies not demonstrating increased cancer incidence (Seiwerth et al., 2021). Both peptides are banned by the World Anti-Doping Agency, citing lack of safety data and potential for abuse in athletic performance enhancement (World Anti-Doping Agency, 2025).

The growth hormone secretagogues (CJC-1295, ipamorelin) occupy a regulatory grey zone. CJC-1295 showed promise in Phase 2 trials, but commercial development ceased following a trial participant’s death, though it was deemed unrelated to the study drug (Teichman et al., 2006). The long-term safety of sustained GH elevation in elderly populations remains incompletely characterized, with concerns about potential cardiovascular effects, glucose dysregulation, and cancer risk, given GH’s biology (Junnila et al., 2013). However, preserving physiological pulsatility with these agents may mitigate the risks associated with continuous exogenous GH administration (Ionescu and Frohman, 2006).

Semax and epitalon have decades of use in Russia with apparently favorable safety profiles, but independent validation and systematic safety surveillance through Western regulatory frameworks are lacking (Khavinson et al., 2012b; Gusev et al., 2018). The absence of serious adverse events in the published Russian literature is encouraging but insufficient for conclusive safety determinations, given publication bias and differences in pharmacovigilance standards.

It is important to emphasize that the investigational peptides discussed in this review (epitalon, GHK-Cu, BPC-157, TB-500, Semax, CJC-1295, and ipamorelin) are not approved by the FDA or equivalent regulatory agencies for the indications described. Their use outside of registered clinical trials carries significant uncertainties regarding product purity, sterility, and dosing consistency, particularly when obtained through compounding pharmacies or unregulated suppliers. Long-term safety surveillance for these agents is absent, and the available evidence is insufficient to constitute clinical guidance. Readers are strongly encouraged to interpret the findings in this review as hypothesis-generating rather than practice-defining, and to refer patients to registered clinical trials where available.

### 7.3 Translational considerations

A persistent challenge across all investigational peptides reviewed here is cross-species translation. Dose scaling from rodent studies to humans is complicated by differences in metabolic rate, body surface area, and lifespan; endpoints used in animal longevity models (e.g., maximum lifespan extension) have no direct human equivalent; and the time scales of animal studies (months) are incommensurable with the decades over which aging interventions would need to operate in humans. Regulatory agencies currently require disease-specific endpoints rather than healthspan measures, further limiting translational pathways.

### 7.4 Current knowledge gaps

Several critical knowledge gaps limit the evidence-based application of gerontological peptides. First, long-term safety data in elderly populations are virtually absent for non-approved peptides. Most animal studies span months rather than years, and human clinical experience derives primarily from short-term trials or uncontrolled clinical use (Wang et al., 2022). Given that aging interventions would require years or decades of use, this represents a fundamental evidence gap.

Second, optimal dosing regimens remain poorly defined for most peptides. Current protocols are based on preclinical extrapolation, anecdotal clinical experience, or single-dose pharmacokinetic studies rather than on systematic dose-finding trials (Muttenthaler et al., 2021). The cyclical dosing patterns common in clinical practice (e.g., 4–6-week cycles with breaks) lack empirical justification beyond theoretical concerns about receptor desensitization or physiological tolerance.

Third, the effects of combination therapy are unexplored. Aging involves multiple interconnected mechanisms, suggesting that multi-peptide protocols targeting complementary pathways might produce synergistic benefits (López-Otín et al., 2023). However, the combination of safety and efficacy remains unstudied, with potential for unexpected interactions or cumulative toxicities.

Fourth, biomarkers for monitoring efficacy and safety are underdeveloped. Beyond specific clinical endpoints (glucose control for tirzepatide, sexual function for bremelanotide), reliable biomarkers indicating beneficial modulation of aging processes are lacking (Kennedy et al., 2014). Telomere length, inflammatory markers, and epigenetic clocks show promise but require validation in peptide intervention contexts.

### 7.5 Potential future developments

The field of gerontological peptides stands at a critical juncture with several promising developments on the horizon. Improved delivery technologies could dramatically enhance clinical utility. Current peptides require frequent injections due to rapid degradation and poor oral bioavailability (Fosgerau and Hoffmann, 2015). Novel formulations (long-acting depot preparations, transdermal systems, intranasal delivery, and oral formulations with absorption enhancers) could improve adherence and patient acceptance (Lau and Dunn, 2018).

Second-generation peptides incorporating chemical modifications to enhance stability, selectivity, and potency are under development. Drug Affinity Complex (DAC) technology used in CJC-1295 exemplifies this approach, extending half-life from minutes to days (Teichman et al., 2006). Similar strategies (PEGylation, cyclization, incorporation of non-natural amino acids) could optimize other gerontological peptides (Muttenthaler et al., 2021).

Precision medicine approaches could identify subpopulations most likely to benefit from specific peptides. Genetic variants affecting GH signaling, telomerase activity, or metabolic pathways might predict responsiveness to corresponding peptides, enabling targeted rather than universal interventions (Kennedy et al., 2014). Integration with comprehensive biomarker panels and aging clocks could guide personalized peptide selection and dosing.

Regulatory pathways specifically designed for aging interventions could accelerate clinical development. Current frameworks require demonstration of efficacy against specific diseases rather than aging itself, creating challenges for peptides with broad healthspan benefits (Kennedy et al., 2014). Novel endpoints (composite measures of functional capacity, disability-free survival, or biological age reduction) could enable approval of genuine anti-aging therapeutics.

Finally, rigorous clinical validation of promising peptides through well-designed trials represents the field’s greatest need. Multi-center, randomized, placebo-controlled trials of epitalon, BPC-157, TB-500, and Semax in elderly populations could definitively establish efficacy and safety, potentially bringing these agents into mainstream gerontological practice (Wang et al., 2022). Such trials would require substantial investment but could yield transformative interventions for healthy aging.

## 8 Conclusions

Therapeutic peptides offer mechanistically diverse approaches to multiple hallmarks of aging, including metabolic dysfunction, telomere attrition, impaired tissue repair, and hormonal decline. FDA-approved agents such as tirzepatide demonstrate the clinical potential of rigorously developed peptide therapeutics for addressing age-related metabolic disease. Other peptides, particularly epitalon for telomere biology, GHK-Cu for dermal aging, and Semax for cognitive aging, show promise but require systematic clinical validation. Complementing these pharmaceutical interventions, natural approaches to age-related hormonal decline offer evidence-based alternatives for individuals preferring or requiring non-pharmacological options. The field faces substantial challenges, including limited long-term safety data, poorly defined optimal dosing, unexplored combination effects, and inadequate regulatory frameworks for aging interventions. Nevertheless, as our understanding of aging biology deepens and peptide technologies advance, these agents, alongside natural interventions, are positioned to play increasingly important roles in extending healthspan and promoting successful aging. Realizing this potential requires commitment to rigorous clinical research, careful attention to safety, and development of appropriate regulatory pathways that balance innovation with patient protection.

## Glossary

ACTH: adrenocorticotropic hormone
Akt-eNOS: Akt-endothelial nitric oxide synthase
BDNF: brain-derived neurotrophic factor
BPC-157: Body Protection Compound-157
cAMP: cyclic adenosine monophosphate
CNS: central nervous system
DAC: Drug Affinity Complex
ED: erectile dysfunction
EGR-1: early growth response protein 1
ERK: extracellular signal-regulated kinase
ERK1/2: extracellular signal-regulated kinase 1/2
FDA: Food and Drug Administration
GH: growth hormone
GHK-Cu: Glycyl-L-histidyl-L-lysine-Copper
GHRH: growth hormone-releasing hormone
GHS-R: growth hormone secretagogue receptor
GHS-R1a: growth hormone secretagogue receptor 1a
GI: gastrointestinal
GIP: glucose-dependent insulinotropic polypeptide
GLP-1: glucagon-like peptide-1
HSDD: hypoactive sexual desire disorder
hTERT: human telomerase reverse transcriptase
IGF-1: insulin-like growth factor-1
MAPK: mitogen-activated protein kinase
MC1R: melanocortin receptor 1
MC3R: melanocortin receptor 3
MC4R: melanocortin receptor 4
MC5R: melanocortin receptor 5
MI: myocardial infarction
MSH: melanocyte-stimulating hormone
NO: nitric oxide
PT-141: bremelanotide (peptide therapeutics-141) ()
TB-500: Thymosin Beta-500
trkB: tropomyosin receptor kinase B
VEGF: vascular endothelial growth factor
VEGFR2: vascular endothelial growth factor receptor-2
WADA: World Anti-Doping Agency

## References

[B1] Alba M. Fintini D. Sagazio A. Lawrence B. Castaigne J. P. Frohman L. A. (2006). Once-daily administration of CJC-1295, a long-acting growth hormone-releasing hormone (GHRH) analog, normalizes growth in the GHRH knockout mouse. Am. J. Physiol. Endocrinol. Metab. 291 (6), E1290–E1294. 10.1152/ajpendo.00201.2006 16822960

[B2] Araj S. K. Brzezik J. Madra-Gackowska K. Szeleszczuk Ł. (2025). Overview of epitalon-highly bioactive pineal tetrapeptide with promising properties. Int. J. Mol. Sci. 26 (6), 2691. 10.3390/ijms26062691 40141333 PMC11943447

[B3] Blackburn E. H. Epel E. S. Lin J. (2015). Human telomere biology: a contributory and interactive factor in aging, disease risks, and protection. Science 350 (6265), 1193–1198. 10.1126/science.aab3389 26785477

[B4] Bolgova O. Shypilova I. Mavrych V. (2025). Natural strategies to optimize estrogen levels in aging women: mini review. Front. Aging 6, 1706117. 10.3389/fragi.2025.1706117 41377590 PMC12685915

[B5] Chang C. H. Tsai W. C. Lin M. S. Hsu Y. H. Pang J. H. S. (2011). The promoting effect of pentadecapeptide BPC 157 on tendon healing involves tendon outgrowth, cell survival, and cell migration. J. Appl. Physiol. 110 (3), 774–780. 10.1152/japplphysiol.00945.2010 21030672

[B6] Dolotov O. V. Karpenko E. A. Inozemtseva L. S. Seredenina T. S. Levitskaya N. G. Rozyczka J. (2006). Semax, an analog of ACTH(4-10) with cognitive effects, regulates BDNF and trkB expression in the rat hippocampus. Brain Res. 1117 (1), 54–60. 10.1016/j.brainres.2006.07.108 16996037

[B7] Erickson K. I. Prakash R. S. Voss M. W. Chaddock L. Heo S. McLaren M. (2010). Brain-derived neurotrophic factor is associated with age-related decline in hippocampal volume. J. Neurosci. 30 (15), 5368–5375. 10.1523/JNEUROSCI.6251-09.2010 20392958 PMC3069644

[B8] Farage M. A. Miller K. W. Elsner P. Maibach H. I. (2013). Characteristics of the aging skin. Adv. Wound Care. 2 (1), 5–10. 10.1089/wound.2011.0356 24527317 PMC3840548

[B9] Fernández-Garza L. E. Guillen-Silva F. Sotelo-Ibarra M. A. Domínguez-Mendoza A. E. Barrera-Barrera S. A. Barrera-Saldaña H. A. (2025). Growth hormone and aging: a clinical review. Front. Aging 6, 1549453. 10.3389/fragi.2025.1549453 40260058 PMC12009952

[B10] Filippenkov I. B. Shpetko Y. Y. Ales D. A. Stavchansky V. V. Denisova A. E. Yuzhakov V. V. (2025). Genes that associated with action of ACTH-Like peptides with neuroprotective potential in rat brain regions with different degrees of ischemic damage. Int. J. Mol. Sci. 26 (13), 6256. 10.3390/ijms26136256 40650034 PMC12249733

[B11] Fosgerau K. Hoffmann T. (2015). Peptide therapeutics: current status and future directions. Drug Discov. Today 20 (1), 122–128. 10.1016/j.drudis.2014.10.003 25450771

[B12] Franceschi C. Campisi J. (2014). Chronic inflammation (inflammaging) and its potential contribution to age-associated diseases. J. Gerontol. A Biol. Sci. Med. Sci. 69 (Suppl. 1), S4–S9. 10.1093/gerona/glu057 24833586

[B13] Frías J. P. Davies M. J. Rosenstock J. Pérez Manghi F. C. Fernández Landó L. Bergman B. K. (2021). Tirzepatide *versus* semaglutide once weekly in patients with type 2 diabetes. N. Engl. J. Med. 385 (6), 503–515. 10.1056/NEJMoa2107519 34170647

[B14] Goldstein A. L. Kleinman H. K. (2015). Advances in the basic and clinical applications of thymosin β4. Expert Opin. Biol. Ther. 15 (Suppl. 1), S139–S145. 10.1517/14712598.2015.1011617 26096726

[B15] Gould L. Abadir P. Brem H. Carter M. Conner-Kerr T. Davidson J. (2015). Chronic wound repair and healing in older adults: current status and future research. Wound Repair Regen. 23 (1), 1–13. 10.1111/wrr.12245 25486905 PMC4414710

[B16] Gusev E. I. Martynov M. Y. Kostenko E. V. Petrova L. V. Bobyreva S. N. (2018). Éffektivnost' semaksa pri lechenii bol'nykh na raznykh stadiiakh ishemicheskogo insul'ta [the efficacy of semax in the tretament of patients at different stages of ischemic stroke]. Zh Nevrol. Psikhiatr Im. S S Korsakova 118 (3. Vyp. 2), 61–68. 10.17116/jnevro20181183261-68 29798983

[B17] Gwyer D. Wragg N. M. Wilson S. L. (2019). Gastric pentadecapeptide body protection compound BPC 157 and its role in accelerating musculoskeletal soft tissue healing. Cell Tissue Res. 377 (2), 153–159. 10.1007/s00441-019-03016-8 30915550

[B18] Harada C. N. Natelson Love M. C. Triebel K. L. (2013). Normal cognitive aging. Clin. Geriatr. Med. 29 (4), 737–752. 10.1016/j.cger.2013.07.002 24094294 PMC4015335

[B19] Ionescu M. Frohman L. A. (2006). Pulsatile secretion of growth hormone (GH) persists during continuous stimulation by CJC-1295, a long-acting GH-releasing hormone analog. J. Clin. Endocrinol. Metab. 91 (12), 4792–4797. 10.1210/jc.2006-1702 17018654

[B20] Jastreboff A. M. Aronne L. J. Ahmad N. N. Wharton S. Connery L. Alves B. (2022). Tirzepatide once weekly for the treatment of obesity. N. Engl. J. Med. 387 (3), 205–216. 10.1056/NEJMoa2206038 35658024

[B21] Junnila R. K. List E. O. Berryman D. E. Murrey J. W. Kopchick J. J. (2013). The GH/IGF-1 axis in ageing and longevity. Nat. Rev. Endocrinol. 9 (6), 366–376. 10.1038/nrendo.2013.67 23591370 PMC4074016

[B22] Kennedy B. K. Berger S. L. Brunet A. Campisi J. Cuervo A. M. Epel E. S. (2014). Geroscience: linking aging to chronic disease. Cell 159 (4), 709–713. 10.1016/j.cell.2014.10.039 25417146 PMC4852871

[B23] Khavinson V. K. Bondarev I. E. Butyugov A. A. (2003). Epithalon peptide induces telomerase activity and telomere elongation in human somatic cells. Bull. Exp. Biol. Med. 135 (6), 590–592. 10.1023/a:1025493705728 12937682

[B24] Khavinson V. K. Linkova N. S. Kvetnoy I. M. Kvetnaia T. V. Polyakova V. O. Korf H. W. (2012a). Molecular cellular mechanisms of peptide regulation of melatonin synthesis in pinealocyte culture. Bull. Exp. Biol. Med. 153 (2), 255–258. 10.1007/s10517-012-1689-5 22816096

[B25] Khavinson V. K. Kuznik B. I. Ryzhak G. A. (2012b). Peptide bioregulators: the new class of geroprotectors. Communication 1. Results of experimental studies. Adv. Gerontol. 25 (4), 696–708. Available online at: https://pubmed.ncbi.nlm.nih.gov/23734519/. 23734519

[B26] Kingsberg S. A. Clayton A. H. Portman D. Williams L. A. Krop J. Jordan R. (2019). Bremelanotide for the treatment of hypoactive sexual desire disorder: two randomized phase 3 trials. Obstet. Gynecol. 134 (5), 899–908. 10.1097/AOG.0000000000003500 31599840 PMC6819021

[B27] Lau J. L. Dunn M. K. (2018). Therapeutic peptides: historical perspectives, current development trends, and future directions. Bioorg Med. Chem. 26 (10), 2700–2707. 10.1016/j.bmc.2017.06.052 28720325

[B28] Lee E. Burgess K. (2025). Safety of intravenous infusion of BPC157 in humans: a pilot study. Altern. Ther. Health Med. 31 (5), 20–24. Available online at: https://pubmed.ncbi.nlm.nih.gov/40131143/ (Accessed January 15, 2026). 40131143

[B29] Lee E. Padgett B. (2021). Intra-articular injection of BPC 157 for multiple types of knee pain. Altern. Ther. Health Med. 27 (4), 8–13. Available online at: https://pubmed.ncbi.nlm.nih.gov/34324435/ (Accessed January 15, 2026). 34324435

[B30] Lee E. Walker C. Ayadi B. (2024). Effect of BPC-157 on symptoms in patients with interstitial cystitis: a pilot study. Altern. Ther. Health Med. 30 (10), 12–17. Available online at: https://pubmed.ncbi.nlm.nih.gov/39325560/ (Accessed January 15, 2026). 39325560

[B31] López-Otín C. Blasco M. A. Partridge L. Serrano M. Kroemer G. (2023). Hallmarks of aging: an expanding universe. Cell 186 (2), 243–278. 10.1016/j.cell.2022.11.001 36599349

[B32] Madeo F. Carmona-Gutierrez D. Hofer S. J. Kroemer G. (2019). Caloric restriction mimetics against age-associated disease: targets, mechanisms, and therapeutic potential. Cell Metab. 29 (3), 592–610. 10.1016/j.cmet.2019.01.018 30840912

[B33] Mansour G. K. Bolgova O. Hajjar A. W. Mavrych V. (2025). Neurovascular dysfunction and glymphatic impairment: an unexplored therapeutic frontier in neurodegeneration. Int. J. Mol. Sci. 26 (24), 11843. 10.3390/ijms262411843 41465272 PMC12733005

[B130] Malavige G. N. Jeewandara C. Ghouse A. Somathilake G. Tissera H. (2021). Changing epidemiology of dengue in Sri Lanka - challenges for the future. PLoS Negl. Trop. Dis. 15 (8), e0009624. 10.1371/journal.pntd.0009624 34411101 PMC8375976

[B34] Mavrych V. Riyas F. Bolgova O. (2025). The role of basolateral amygdala and medial prefrontal cortex in fear: a systematic review. Cureus 17 (1), e78198. 10.7759/cureus.78198 40026920 PMC11870299

[B36] Molinoff P. B. Shadiack A. M. Earle D. Diamond L. E. Quon C. Y. (2003). PT-141: a melanocortin agonist for the treatment of sexual dysfunction. Ann. N. Y. Acad. Sci. 994, 96–102. 10.1111/j.1749-6632.2003.tb03167.x 12851303

[B37] Muttenthaler M. King G. F. Adams D. J. Alewood P. F. (2021). Trends in peptide drug discovery. Nat. Rev. Drug Discov. 20 (4), 309–325. 10.1038/s41573-020-00135-8 33536635

[B38] Palmer A. K. Jensen M. D. (2022). Metabolic changes in aging humans: current evidence and therapeutic strategies. J. Clin. Invest 132 (16), e158451. 10.1172/JCI158451 35968789 PMC9374375

[B39] Pickart L. Margolina A. (2018). Regenerative and protective actions of the GHK-Cu peptide in the light of the new gene data. Int. J. Mol. Sci. 19 (7), 1987. 10.3390/ijms19071987 29986520 PMC6073405

[B40] Pickart L. Vasquez-Soltero J. M. Margolina A. (2015). GHK peptide as a natural modulator of multiple cellular pathways in skin regeneration. Biomed. Res. Int. 2015, 648108. 10.1155/2015/648108 26236730 PMC4508379

[B41] Raun K. Hansen B. S. Johansen N. L. Thøgersen H. Madsen K. Ankersen M. (1998). Ipamorelin, the first selective growth hormone secretagogue. Eur. J. Endocrinol. 139 (5), 552–561. 10.1530/eje.0.1390552 9849822

[B42] Rosenstock J. Wysham C. Frías J. P. Kaneko S. Lee C. J. Fernández Landó L. (2021). Efficacy and safety of a novel dual GIP and GLP-1 receptor agonist tirzepatide in patients with type 2 diabetes (SURPASS-1): a double-blind, randomised, phase 3 trial. Lancet 398 (10295), 143–155. 10.1016/S0140-6736(21)01324-6 34186022

[B43] Seiwerth S. Milavic M. Vukojevic J. Gojkovic S. Krezic I. Vuletic L. B. (2021). Stable gastric pentadecapeptide BPC 157 and wound healing. Front. Pharmacol. 12, 627533. 10.3389/fphar.2021.627533 34267654 PMC8275860

[B44] Shypilova I. Bolgova O. Mavrych V. (2026). Integrative natural approaches for age-related testosterone decline: a synergistic framework combining exercise, nutrition, and bioactive compounds. Cureus 18 (1), e101276. 10.7759/cureus.101276 41674746 PMC12887910

[B45] Sosne G. Kleinman H. K. (2015). Primary mechanisms of thymosin β4 repair activity in dry eye disorders and other tissue injuries. Invest Ophthalmol. Vis. Sci. 56 (9), 5110–5117. 10.1167/iovs.15-16890 26241398

[B46] Teichman S. L. Neale A. Lawrence B. Gagnon C. Castaigne J. P. Frohman L. A. (2006). Prolonged stimulation of growth hormone (GH) and insulin-like growth factor I secretion by CJC-1295, a long-acting analog of GH-releasing hormone, in healthy adults. J. Clin. Endocrinol. Metab. 91 (3), 799–805. 10.1210/jc.2005-1536 16352683

[B47] Thomas M. K. Nikooienejad A. Bray R. Cui X. Wilson J. Duffin K. (2021). Dual GIP and GLP-1 receptor agonist tirzepatide improves beta-cell function and insulin sensitivity in type 2 diabetes. J. Clin. Endocrinol. Metab. 106 (2), 388–396. 10.1210/clinem/dgaa863 33236115 PMC7823251

[B48] Toogood A. A. O'Neill P. A. Shalet S. M. (1996). Beyond the somatopause: growth hormone deficiency in adults over the age of 60 years. J. Clin. Endocrinol. Metab. 81 (2), 460–465. 10.1210/jcem.81.2.8636250 8636250

[B49] Ungvari Z. Tarantini S. Kiss T. Wren J. D. Giles C. B. Griffin C. T. (2018). Endothelial dysfunction and angiogenesis impairment in the ageing vasculature. Nat. Rev. Cardiol. 15 (9), 555–565. 10.1038/s41569-018-0030-z 29795441 PMC6612360

[B50] U.S. Food and Drug Administration (2026a). Mounjaro (tirzepatide) injection prescribing information. Available online at: https://www.accessdata.fda.gov/drugsatfda_docs/label/2022/215866s000lbl.pdf (Accessed January 3, 2026).

[B51] U.S. Food and Drug Administration (2026b). Zepbound (tirzepatide) injection prescribing information. Available online at: https://www.accessdata.fda.gov/drugsatfda_docs/label/2023/217806s000lbl.pdf (Accessed January 3, 2026).

[B52] U.S. Food and Drug Administration. (2026c). Zepbound prescribing information. Available online at: https://www.accessdata.fda.gov/drugsatfda_docs/label/2024/217806s003lbl.pdf (Accessed January 3, 2026).

[B53] U.S. Food and Drug Administration (2026d). Vyleesi (bremelanotide) injection prescribing information. Available online at: https://www.accessdata.fda.gov/drugsatfda_docs/label/2019/210557lbl.pdf (Accessed January 3, 2026).

[B54] Wang X. Liu L. Qi L. Lei C. Li P. Wang Y. (2021). A first-in-human, randomized, double-blind, single- and multiple-dose, phase I study of recombinant human thymosin β4 in healthy Chinese volunteers. J. Cell Mol. Med. 25 (17), 8222–8228. 10.1111/jcmm.16693 34346165 PMC8419156

[B55] Wang L. Wang N. Zhang W. Cheng X. Yan Z. Shao G. (2022). Therapeutic peptides: current applications and future directions. Signal Transduct. Target Ther. 7 (1), 48. 10.1038/s41392-022-00904-4 35165272 PMC8844085

[B56] World Anti-Doping Agency (2025). World anti-doping code. International Standard Prohibited List. Available online at: https://www.wada-ama.org/en/prohibited-list (Accessed January 3, 2026).

